# Estimating Linkage Disequilibrium and Effective Population Size Across Generations in Holstein Cattle

**DOI:** 10.1002/vms3.70684

**Published:** 2025-11-14

**Authors:** Ronak Salehi, Arash Javanmard, Mahdi Mokhber, Sadegh Alijani

**Affiliations:** ^1^ Department of Animal Science Faculty of Agricultural University of Tabriz Tabriz Iran; ^2^ Department of Animal Science Faculty of Agriculture Urmia University Urmia Iran

**Keywords:** effective population size, genetic diversity, inbreeding, linkage disequilibrium, SNP markers

## Abstract

**Background:**

Understanding the structure of linkage disequilibrium (LD) and accurately estimating the effective population size (Ne) are crucial for maintaining genetic diversity and ensuring population survival. These metrics are vital for decision‐making in conservation genetics and breeding programs.

**Objectives:**

This study aimed to analyse the LD structure and estimate Ne in global Holstein cattle populations to assess genetic diversity and population dynamics.

**Methods:**

Genomic data from 2127 cows across eight countries (Poland, Sweden, Ireland, Iran, France, China, Canada and the Netherlands) were analysed. Data quality control was performed using PLINK 1.9. Adjusted *R*‐squared (*r* ^2^) values for SNP markers up to 38 Mbp and Ne values from ancestral generations to the present were calculated using SNeP 1.1.

**Results:**

LD values decreased nonlinearly with increasing physical distance, ranging from 0.102–0.320 at <25 kbp to 0.007–0.059 at 38 Mbp. Ne values have declined significantly since 2000 generations ago, with a sharp reduction from 70 to 10 generations ago. However, the decline slowed in the last 10 generations, with slight increases in some populations. Current Ne values range from 74 (French Holstein) to 171 (Polish Holstein). The rapid decline in Ne is attributed to the intensive use of limited superior bulls, reducing genetic diversity.

**Conclusions:**

The recent slowdown in Ne decline and slight increases in some populations may reflect improved breeding strategies, including genetic material importation. These findings highlight the importance of managing genetic diversity and mitigating inbreeding effects in Holstein cattle populations. Effective breeding programs are essential to sustain genetic health, productivity and long‐term adaptability in commercial dairy cattle.

## Graphical abstract

This study reveals a significant historical decline in effective population size (Ne) in Holstein cattle due to intensive breeding, with recent stabilization reflecting improved genetic management. Maintaining genetic diversity is vital for sustainable dairy cattle breeding programs.

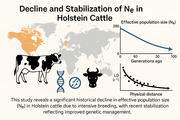

## 1 Introduction

In an infinitely large population and in the absence of systematic factors that alter allele frequencies (e.g., mutation, selection and migration), the allele and genotype frequencies of a genetic locus remain constant over time (Crow 1999). Conversely, in cases where the population size is small, the random sampling process changes in these genetic characteristics because only a limited number of gametes will have a chance of being present in the next generation (Wright 1931). The effectiveness of random sampling and the level of changes in the genetic characteristics of the population depend on the concept of the effective population size (Ne) (Crow and Kimura 1970). The concept of Ne was first introduced by Wright in 1931 and later developed by other researchers (Caballero 1994; Charlesworth 2009; Crow 2017). Random changes in genetic traits occur more slowly in populations with high Ne than in populations with low Ne (Hayes et al. 2003). Ne levels, along with systematic forces that change allele frequencies (e.g., mutation, selection, migration and recombination), determine the amount and pattern of genetic diversity present in a population. Thus, Ne is highly important for describing the amount and pattern of a population's observed genetic diversity, interpreting the evolutionary mechanisms involved in the formation of diversity in natural populations and understanding population evolution and recombination (Barton and Charlesworth 1998). In addition, Ne helps predict the genetic diversity of neutral loci, the probability of fixation of beneficial alleles or elimination of alleles (Robertson 1961) and the fitness and survival of a small population (Lynch et al. 1995). Ne is an essential parameter in the evolution and biological conservation of populations (Wang 2005). Knowing Ne facilitates the design of artificial selection programs in plant and animal breeding (Caballero et al. 1994) and the efficient management of populations of endangered species (Wang 2004). Accordingly, there is great interest in identifying Ne in natural and artificial populations among researchers in population genetics, quantitative genetics, evolutionary biology and conservation (Barton and Charlesworth 1998).

The linkage disequilibrium (LD) pattern is a powerful indicator of population genetic processes, and understanding this pattern can help determine Ne and know the demographic history of a population (Qanbari 2020). Estimates obtained for the Ne in the past of any population can be important in determining information such as population growth rates and the occurrence of influential events such as bottlenecks in the past of the population (Browning et al. 2018; Palamara et al. 2012). Using simulation, Hayes et al. (2003) demonstrated that LD at short distances is a function of Ne in more distant previous generations, whereas at long distances, it reflects the state of the population in recent generations. Hence, by determining the amount of LD for different distances, Ne can be identified as a key factor for different generations in the past. Two statistics, *r* ^2^ and *D*′, are used to compute LD between two marker loci (Bohmanova et al. 2010). Among them, the *r* ^2^ statistic is less affected by allele frequency and sample size compared to *D*′. Therefore, it is a key statistic in determining LD (Bohmanova et al. 2010). Recent advances in sequencing technologies have facilitated obtaining information on the LD structure at the genome level, and this information is ideal for estimating Ne values in human and domestic animal populations compared to other methods of estimating the effective population size (Ne) (Kijas et al. 2012).

Studies have investigated linkage disequilibrium (LD) structure and effective population size (Ne) in multiple livestock species, with reported Ne estimates ranging from 33 to 153 in dairy cattle (Rodríguez‐Ramilo et al. 2015; Doekes et al. 2018; Makanjuola, Maltecca, et al. 2020; Chhotaray et al. 2021; Dlamini et al. 2022; Sarviaho et al. 2023), 43 to 212 in buffaloes (Mokhber, Moradi Shahre Babak, et al. 2019; Rahimmadar et al. 2021; Strillacci et al. 2021), 197 to 838 in sheep (Ramljak et al. 2024), 243 to 593 in goats (Chalebgwa et al. 2025), 21 to 72 in pigs (Zorc et al. 2022), 27 to 54 in horses (Bazvand et al. 2024) and 15 to 189 in poultry (Zhang et al. 2020).

The present study aims to use genomic information to determine the LD structure and estimate the effective population size (Ne) of Holstein cattle populations worldwide. It is hoped that the results of this study will be useful in analysing population structures, and, if necessary, policies to preserve genetic diversity can be made using this information.

## 2 Materials and Methods

### 2.1 Genomic Data and Quality Control (QC)

In the current study, genomic data from 2127 Holstein cattle representing 8 countries (Poland, Sweden, Ireland, Iran, France, China, Canada and the Netherlands) were used; the details are provided in Table 1. These data were obtained from the Dryad and WIDDE databases (Sempéré et al. 2015) or through direct correspondence with researchers. Although the dataset comprises nine genomic data files, they originate from eight countries. For the French Holstein population, two low‐density (LD, ∼54 K SNPs) datasets—consisting of 63 and 30 animals, respectively—were merged due to their similar density and common origin. In contrast, the high‐density (HD, ∼777 K SNPs) dataset from France, despite its shared origin, was kept separate to preserve the higher marker density, which is particularly valuable for studies focused on fine‐scale genomic diversity and structure. Therefore, only the LD datasets were pooled, whereas the HD dataset was analysed independently.

**TABLE 1:** Genomic data of nine datasets used Holstein populations related to eight countries, including Poland, Sweden, Ireland, Iran, France, China, Canada and the Netherlands.

Row no.	Mark	Country	Sample no.	Quick access address—genomic array	Relevant article
1	CAN	Canada	37	—	—
2	CHN	China	1508	—	—
3	NLD	The Netherlands	20	https://datadryad.org/stash/dataset/doi:10.5061/dryad.th092	Decker et al. (2015)
4	FRA_LD	France	93	WIDD and https://doi.org/10.1371/journal.pone.0013038 https://doi.org/10.1371/journal.pone.0005350 and WIDD Illumina_ BovineSNP50v1	Gautier et al. (2010), Matukumalli et al. (2009)

5	FRA_HD	France	60	http://widde.toulouse.inra.fr/widde/widde/main.do;jsessionid=EB8D8557C0E6C34E3040E06D29FDA729?module=cattle and WIDD umina.com/applications/agriculture.ilmn Illumina BovineHD	Project: ILLUMINA_HD
6	IRI	Iran	43	Illumina_ BovineSNP50v2	Shakeri et al. (2021)
7	IRL	Ireland	145	Data available from the Dryad Digital Repository: doi: 10.5061/dryad.519bm	Bermingham et al. (2014)
8	POL	Poland	198	Illumina_ BovineSNP50v1	Requested
9	SWE	Sweden	23	—	Salehi et al. (2023)
Total	—	—	2127	—	—

Abbreviation: HD, high‐density.

The selection of these specific Holstein populations was primarily based on the availability of high‐quality genomic data meeting the minimum threshold required for robust analysis. Moreover, the dataset encompasses a broad geographical distribution—including countries from North America (Canada), Western and Eastern Europe (France, the Netherlands, Ireland, Poland and Sweden) and Asia (Iran and China)—providing a diverse representation of global Holstein populations with different breeding programs and genetic backgrounds.

QC and data filtration were performed using PLINK Ver1.9 software (Purcell et al. 2007). Animals and loci with more than 5% missing genotypes—measured as call rate of individuals (CR_IND_) and call rate of SNPs (CR_SNP_)—as well as monomorphic genotypes, genotypes with minor allele frequency (MAF) less than 5%, and SNPs deviating from Hardy–Weinberg equilibrium were removed. Details on the animals, genomic data and QC thresholds are provided in Table 2.

**TABLE 2:** Descriptive statistics for the studied Holstein populations.

Threshold	Number of animal before QC	Number of animal after QC	Filterd SNP					SNP number after QC
Populations			SNP number before QC	MAF < 5%	SNP call rate <0.05	MIND < 0.05	HWE < 10^−6^	
Polish	198	198	54,001	11,124	198	0	157	41,794
Sweden	23	23	120,993	0	0	0	0	120,993
Ireland	145	145	617,885	44,027	0	0	157	573,701
Iran	43	43	47,843	3415	1344	0	7	43,077
France_HD	60	60	733,454	154,396	3046	0	58	575,954
France_LD	93	93	51,998	10,626	2277	0	0	39,095
China	1508	1508	52,886	11,903	0	0	332	40,651
Canada	37	37	53,218	6422	5934	0	476	40,386
The Netherlands	20	20	42,799	6416	43	0	0	36,340

Abbreviations: HD, high‐density; MAF, minor allele frequency; QC, quality control.

Additionally, SNPs with unkown position and on sex chromosems were eliminated from filtered data. Considering the previously published studies on the genetic diversity of Holstein populations, the overlapping data were not presented or analysed in this investigation. In addition, the data from different series were sequenced using different arrays from Illumina and Genseek in order to maintain the maximum number of informative SNP markers. Thus, genomic information related to each population was utilized separately, but with the same screening threshold, to determine the structure of LD and estimate the effective population size (Ne).

**FIGURE 1:**
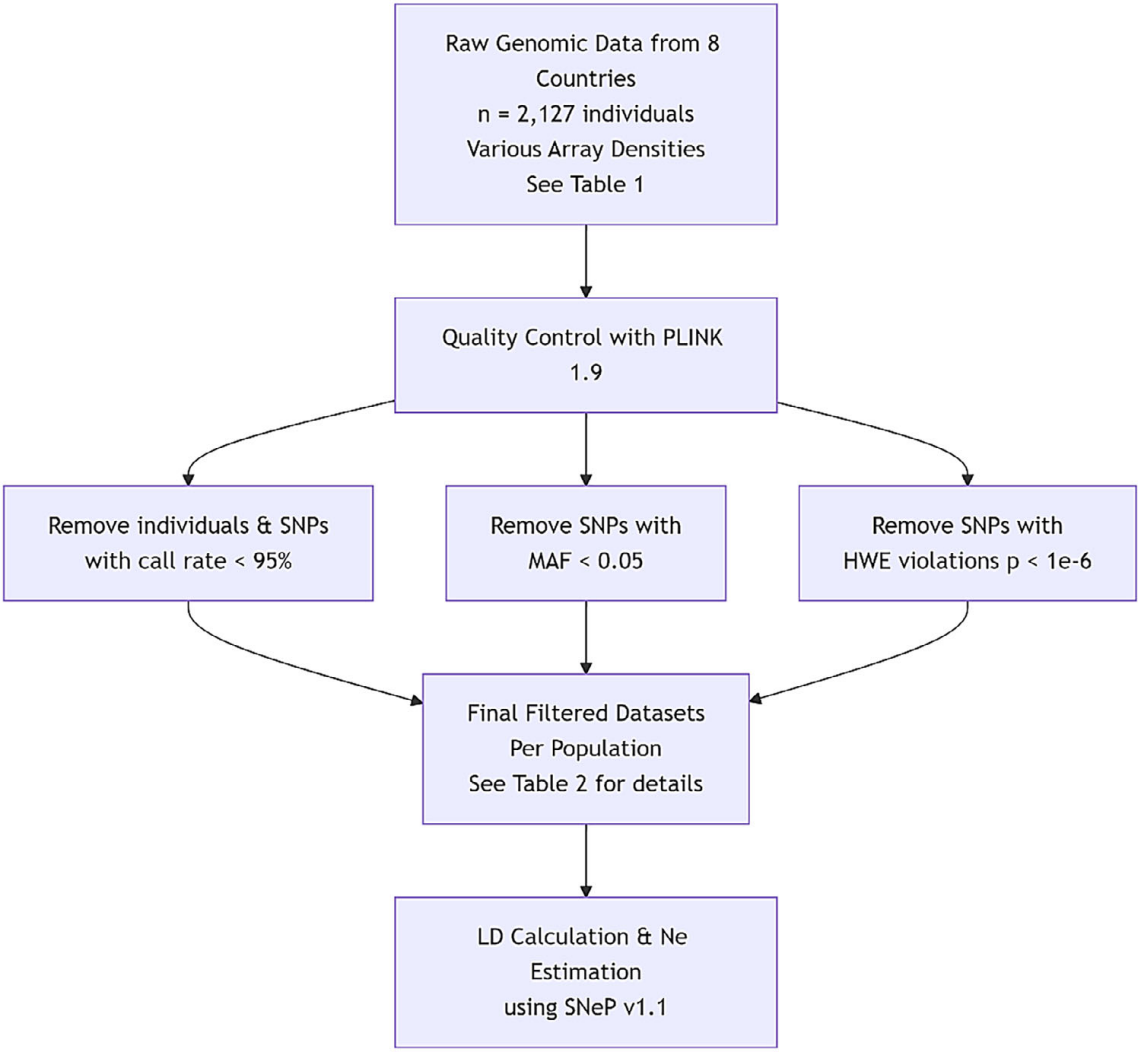
Schematic workflow of the genomic analysis. The diagram illustrates the key steps from data acquisition to result generation, including quality control (QC), linkage disequilibrium (LD) calculation and effective population size (Ne) estimation.

A schematic overview of the complete analytical workflow from raw data processing to final results is presented in Figure 1.

### 2.2 LD Structure and Effective Population Size (Ne)

The calculation of *r* ^2^ values and the estimation of Ne for past and present generations were conducted using the *r* ^2^ pairwise information via SNeP Ver1.1 software over specified distances (up to 40 Mb). The selection of up to 40 Mb distance threshold for calculating pairwise linkage disequilibrium (*r* ^2^) follows established practices in livestock genomics, allowing assessment of both short‐ and long‐range LD decay. Long‐range LD provides critical information about recent recombination events and is essential for estimating effective population size (Ne) in recent generations (Hayes et al. 2003; Tenesa et al. 2007). Previous studies in cattle have similarly used thresholds between 30 and 50 Mb to capture LD patterns comprehensively (Sánchez et al. 2014; Bohmanova et al. 2010; Espigolan et al. 2013). Additionally, Corbin et al. (2012) highlighted the importance of including long‐range LD in demographic inference. Thus, the 40 Mb limit represents a balance between biological relevance, computational feasibility and alignment with prior literature, a clarification now added to the manuscript. Thresholds included for SNeP Ver1.1 were ‐mindist 10,000; ‐maxdist 40,000,000; ‐maxsnp 100,000; ‐binwidth 50,000 and ‐numBINS 40.

The linkage disequilibrium (LD) values, corrected for each population, were used to estimate the effective population size (Ne) according to the following equation (Sved 1971):

$$\mathrm{Ne}=\left(\frac{1}{4c}\right)\left(1-\frac{1}{r^{2}}\right)$$

where Ne represents the effective population size at a given generation (*T*), *r* ^2^ is the average pairwise LD for a specific genomic distance, and *c* is the recombination rate in Morgans (approximated as 1 cM ≈ 1 Mb), as proposed by Tenesa et al. (2007) and Villa‐Angulo et al. (2009). The corresponding generation number (*T*) was calculated using the following equation (Hayes et al. 2003):

$$T=\frac{1}{2c}$$

This analytical framework enables the estimation of Ne at different historical time points, with *T* inversely related to the recombination distance. Convergence in our analysis was defined as the point where Ne values stabilized across adjacent genomic distance bins, such that further increases in bin size did not meaningfully affect the estimated value. This ensures that Ne estimates reflect a stable LD decay pattern rather than noise due to sparse marker coverage.

### 2.3 Methodological Considerations and Limitations

To ensure transparency and reproducibility, several methodological limitations should be acknowledged. First, there was considerable imbalance in sample sizes across populations—for example, 1508 individuals from China versus only 23 from Sweden. Such disparity can introduce sampling bias, particularly in the estimation of linkage LD and Ne. Although LD estimates are generally robust when calculated from a large number of SNP pairs, small population sizes may lead to increased variance in LD values, especially over longer genomic distances, thus affecting recent Ne estimates (Barbato et al. 2015; Corbin et al. 2012). Second, the use of different SNP genotyping platforms (e.g., 54 vs. 777 K arrays) may influence LD resolution and Ne accuracy. HD arrays enable finer scale detection of LD decay, providing better insight into recent demographic changes, whereas lower density platforms may miss short‐range LD signals (Corbin et al. 2012). Third, the approximation of physical to genetic distance (i.e., 1 Mb ≈ 1 cM) is a simplification commonly used in genomic studies (Tenesa et al. 2007; Villa‐Angulo et al. 2009), but this relationship can vary across genomic regions and between breeds due to heterogeneity in recombination rates. Therefore, although this assumption facilitates comparison across populations, it may introduce minor biases in the estimation of recombination distance and, subsequently, Ne. These factors—unequal sampling, marker density, SNP array type and distance approximations—have been taken into consideration when interpreting the results and are now explicitly discussed to improve the clarity and robustness of the study.

## 3 Results and Discussion

### 3.1 Linkage Disequilibrium Structure of Holstein Populations

**FIGURE 2:**
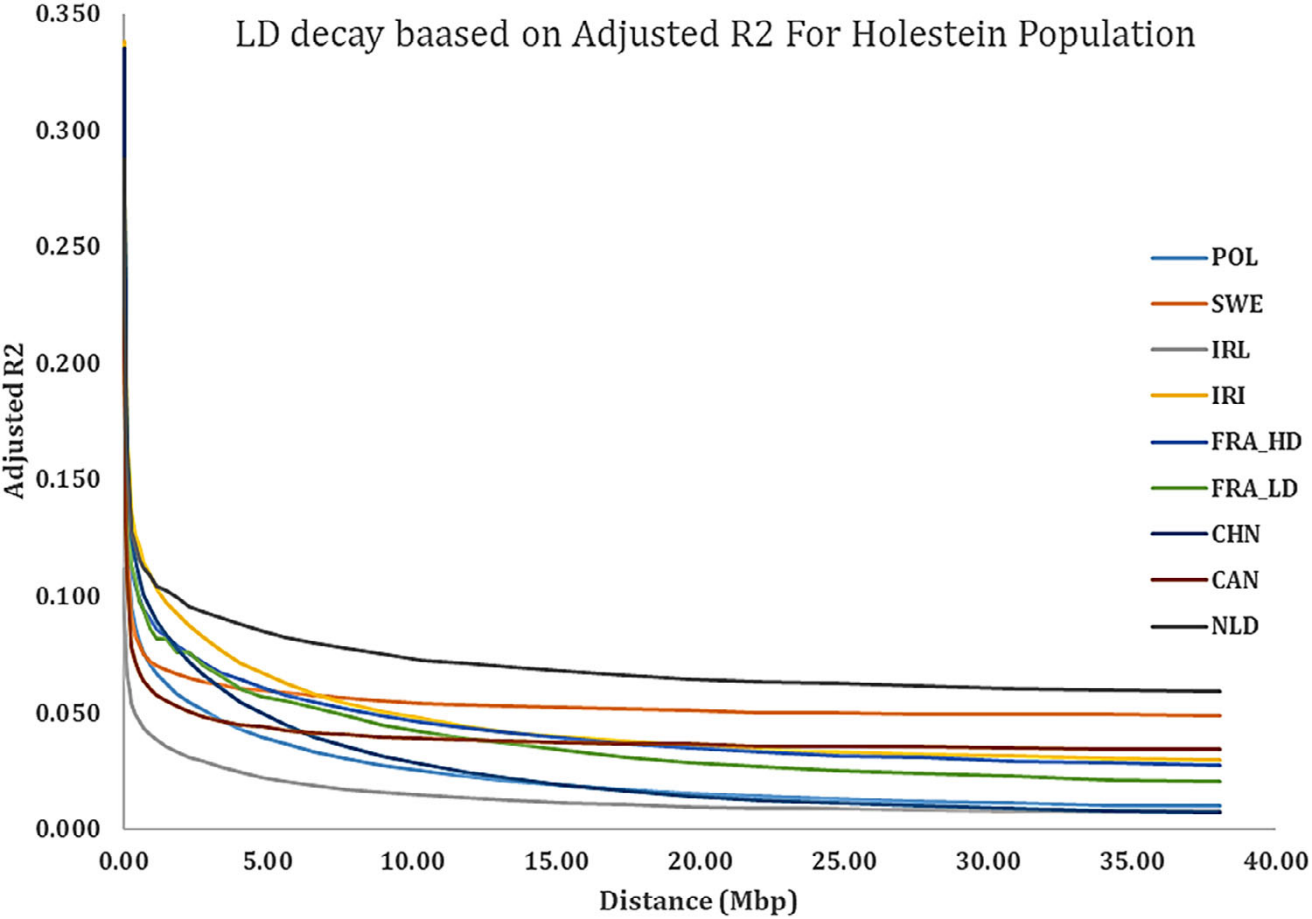
Average LD decay over physical distance from 10 kbp to 40 Mbp for studied Holstein populations. HD, high‐density.

**FIGURE 3:**
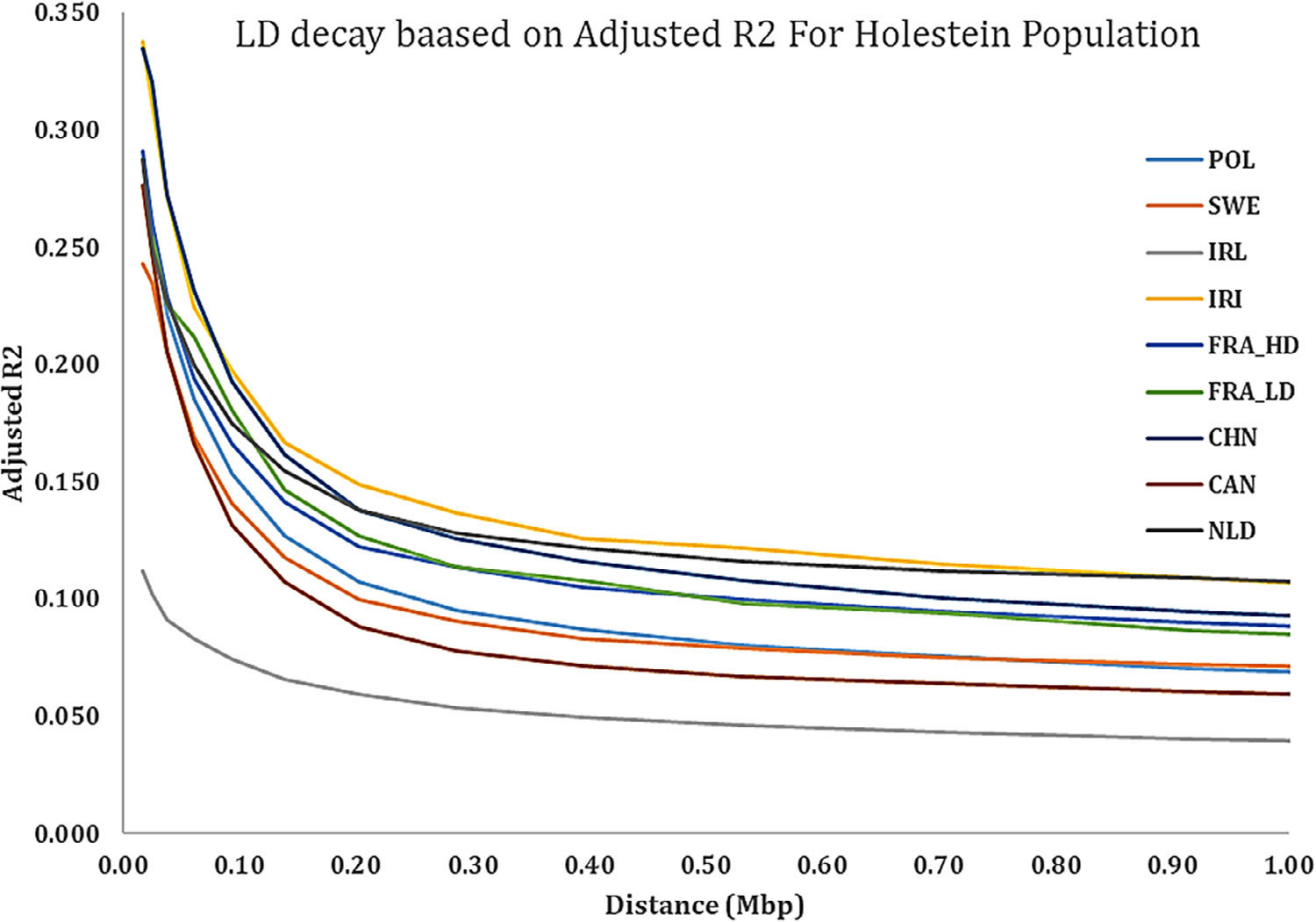
Average LD decay over physical distance from 10 kbp to 1 Mbp for studied Holstein populations. HD, high‐density.

As summarized in Table 3 and illustrated in the LD decay curves (Figures 2 and 3), the adjusted *r* ^2^ values exhibited a characteristic nonlinear decrease with increasing physical distance, spanning from short intervals (<25 kbp) to long‐range distances (up to 38 Mbp). This analysis revealed substantial variability in LD patterns across the studied Holstein populations. At short distances (<25 kbp), the Polish and both French populations showed moderate LD levels (∼0.25–0.26), whereas Iran and China displayed notably higher LD (0.311 and 0.320, respectively). Ireland, in contrast, had the lowest short‐distance LD (0.102). At the maximum distance analysed (38 Mbp), LD values decayed considerably across all populations. The Netherlands and Sweden retained the highest long‐range LD (0.059 and 0.049, respectively), whereas China and Ireland showed the most rapid decay (0.007 and 0.008, respectively). The complete profile of LD decay for each population is detailed in Table 3 and visualized in Figures 2 and 3.

**TABLE 3:** Comparative overview of key genetic parameters in the studied Holstein populations.

Population (code)	SNP array density (K)	Sample size	LD (*r* ^2^) at <25 kbp	LD (*r* ^2^) at ∼1 Mbp	Current Ne (Gen. 1)	Ne 10 generations ago
Poland (POL)	54	198	0.259	0.058	171	160
Sweden (SWE)	777	23	0.235	0.100	155	365
Ireland (IRL)	777	145	0.102	0.036	98	248
Iran (IRI)	54	43	0.311	0.103	121	125
France (HD)	777	60	0.261	0.083	74	127
France (LD)	54	93	0.254	0.082	82	121
China (CHN)	54	1508	0.320	0.101	91	107
Canada (CAN)	54	37	0.247	0.064	115	348
The Netherlands (NLD)	54	20	0.249	0.100	89	161

Abbreviation: HD, high‐density.

Linkage disequilibrium (LD) is a fundamental genomic property that reflects the non‐random association of alleles at different loci and provides critical insight into population history, effective population size (Ne), selection intensity and recombination dynamics (Hayes et al. 2003; Qanbari et al. 2010). The nonlinear decay of adjusted *r* ^2^ values with increasing physical distance between SNPs, as observed in our results (Figures 2 and 3), is a hallmark of structured, recombining genomes, especially under artificial selection pressure such as in modern dairy cattle breeding programs.

Our findings showed a sharp decline in LD from short distances (e.g., <25 kbp) to long‐range distances (up to 38 Mbp), with *r* ^2^ values dropping from as high as 0.320 in the Chinese population to as low as 0.007 in the same population at the longest range. This substantial decay across populations indicates the presence of relatively short haplotype blocks, a feature commonly observed in highly recombining and genetically diverse populations (Flury et al. 2010; Kim et al. 2022).

Populations like Iran (IRI) and China (CHN) exhibited relatively higher LD at shorter distances, which may indicate either a more recent bottleneck, lower effective population size or more intensive within‐population selection. In contrast, Ireland (IRL) and Sweden (SWE) showed lower LD at short distances and flatter decay curves, suggesting either greater historical recombination, more diverse ancestral genetic backgrounds or the impact of structured breeding schemes with broader genetic bases. Similar patterns were reported by de Roos et al. (2008) and Makina et al. (2015), where outbred or admixed populations like Jersey or Nguni cattle showed more rapid LD decay compared to more homogeneously selected Holstein populations.

Interestingly, the difference in LD between the France_HD and France_LD populations—despite both being Holstein and genotyped using HD SNP arrays—suggests that genotyping platform, data filtering stringency and imputation accuracy may significantly influence LD estimation (Wiggans et al. 2012). HD arrays typically provide more accurate LD estimation, especially for shorter distances, but minor variations in SNP call rates or population stratification could also explain subtle differences observed in our dataset. Beyond overall LD structure and Ne dynamics, SNP marker density is known to affect the resolution of LD decay curves and the accuracy of Ne estimation. Higher marker densities can improve LD detection at shorter distances and yield more precise Ne estimates (Sarviaho et al. 2023; Ramljak et al. 2024).

From a functional genomics perspective, the rapid LD decay observed in most populations has important implications. For instance, QTL mapping or GWAS in populations like IRL or SWE would require denser marker coverage or higher imputation accuracy due to weaker long‐range LD. In contrast, the more extended LD observed in CHN or IRI populations may permit broader QTL detection windows but at the cost of resolution and potential false positives. Thus, population‐specific LD profiles are critical not only for association studies but also for genomic prediction, especially in multi‐breed or cross‐country evaluation settings (Pryce et al. 2014).

Furthermore, the variation in LD structure among countries reflects their breeding history and selection regimes. Holstein populations in countries with intensive genomic selection programs (e.g., Canada, the Netherlands and France) may exhibit elevated LD in certain regions due to selective sweeps and strong artificial selection for production traits (Boichard et al. 2012; VanRaden et al. 2009). The long‐term implementation of genomic selection has been shown to reshape haplotype diversity and reduce effective recombination rates locally, which may explain regional LD elevations in some of these populations (Daetwyler et al. 2014).

In summary, our results are in agreement with the general consensus in the literature: LD in dairy cattle decays rapidly with increasing physical distance, but the rate and extent of this decay vary substantially across populations due to differences in selection intensity, demographic history, effective population size and genotyping strategies. These findings reinforce the need for population‐specific models in genomic selection, GWAS and imputation pipelines, especially in globally distributed Holstein populations where regional genomic architectures may differ.

The analysis of LD structure and decay in French Holstein populations using two SNP densities (54 and 777 K) revealed differences ranging from 0 to 0.018. At longer genomic distances, LD values were higher in the HD dataset, whereas at shorter distances, the differences diminished. To evaluate whether these differences were due to marker density or inherent genetic variation, the 700 K dataset was downsampled to match the 54 K array. The results from the reduced‐density data were fully consistent with those from the original 700 K dataset, indicating that the observed differences were primarily due to genetic differences between populations rather than marker density.

### 3.2 Impact of Marker Density on LD Estimation

The density of SNP markers directly affects the accuracy and resolution of LD estimation. Higher SNP density enables more precise detection of LD, especially over short distances, thereby offering a clearer picture of the population's genomic architecture. Conversely, lower marker density may fail to capture weak or short‐range LD, particularly in regions with high recombination rates or complex genomic structure (Khatkar et al. 2008; Qanbari and Simianer 2014). A key limitation of using low‐density data is that sparse marker spacing can lead to unstable or less accurate LD estimates, reducing the power to detect QTLs or trait‐associated variants (Wientjes et al. 2013). Lower density may also impair estimates of LD block size, selection of tagSNPs and the efficiency of genomic prediction models.

Nonetheless, HD genotyping is costly and computationally demanding. A practical alternative can be the combined use of panels with different densities along with genotype imputation methods, which balance accuracy and resource use (VanRaden et al. 2011).

Considering that genetic diversity and biological conservation depend on Ne (Wang 2005), accurate estimation of population size is highly essential to prevent diversity decline and maintain population survival and is typically the main basis for genetic conservation decisions. According to FAO (1992), when Ne equals 25, 50, 125, 250 and 500, genetic diversity will decrease by about 18%, 10%, 4%, 1.6% and 0.8% over 10 generations, respectively. Furthermore, evidence obtained from 1980 confirms that Ne >100 individuals is necessary for a limited decrease in fitness (<10%) over 5 generations for wild populations, whereas this amount should be >1000 individuals to maintain the evolutionary potential of the population in the long term (Frankham et al. 2010). Moreover, Meuwissen (2009) revealed that if Ne is >100 individuals, the population will be viable in terms of genetic diversity in the long term.

**FIGURE 4:**
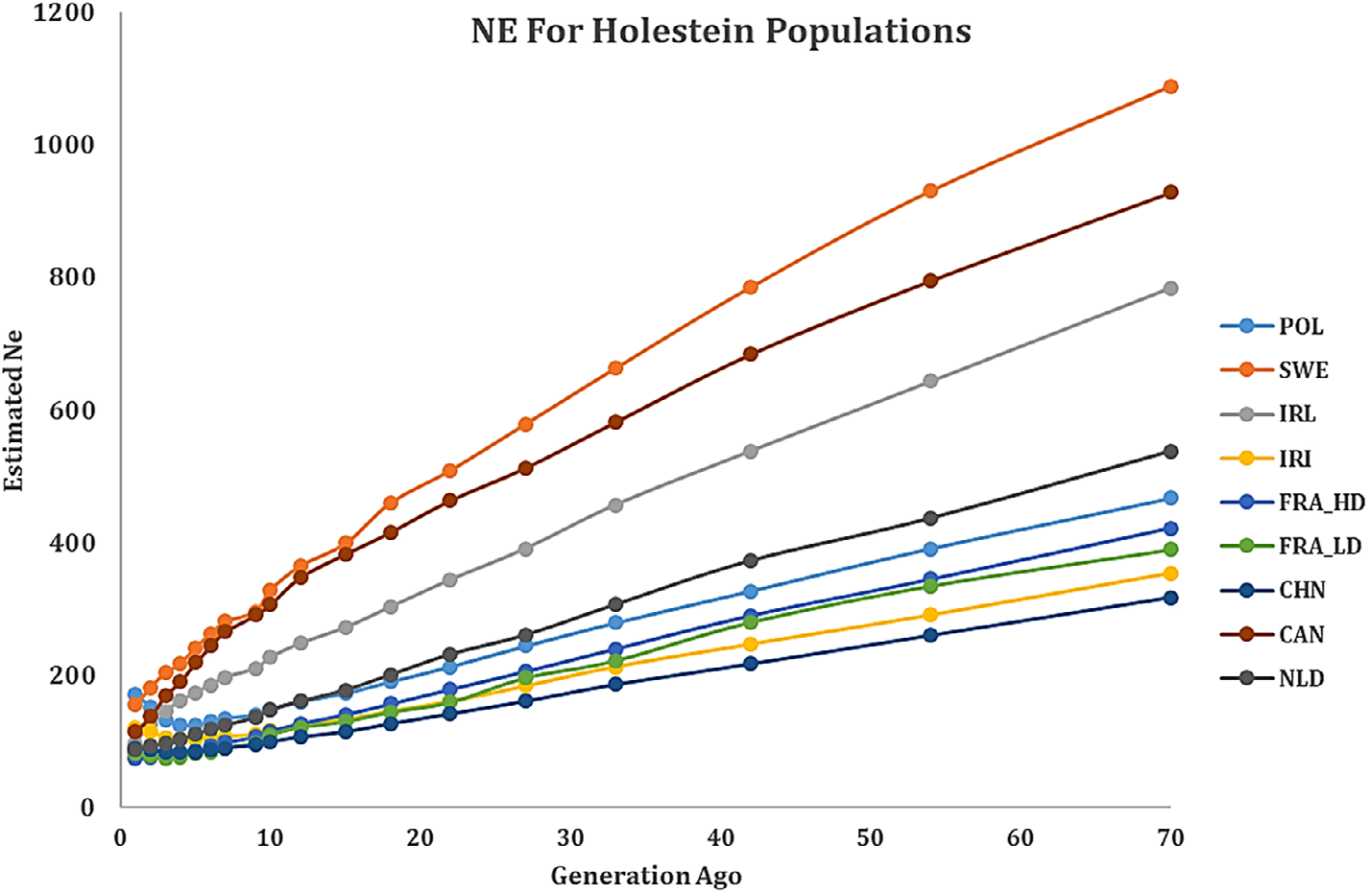
Estimated Ne for Holstein populations from 70 generations ago to recent based on corrected *r* ^2^. HD, high‐density.

**FIGURE 5:**
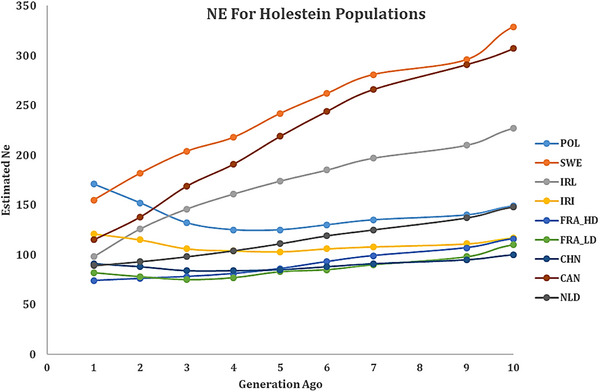
Estimated Ne for Holstein populations from 10 generations ago to recent based on corrected *r* ^2^. HD, high‐density.

One of the most common methods for estimating Ne is to use LD information, which is dependent on the availability of a large amount of genetic marker data. Effective population size (Ne) was estimated using the SNeP software, which calculates Ne based on linkage disequilibrium patterns derived from genome‐wide SNP data (Barbato et al. 2015; Burren et al. 2014). This method allows for population‐scale demographic inference while incorporating genomic parameters such as recombination and mutation rates. Estimating Ne over historical timeframes provides insights into the demographic history of Holstein cattle populations. In this study, Ne was estimated from 4000 generations ago to the present. However, due to lower SNP density in some populations, Ne values for time points beyond 2000 generations ago could not be reliably calculated at short inter‐marker distances (<17 kbp). Therefore, comparisons between populations are based on estimates from 2000 generations ago to the present, where the data are considered more robust. For a visual examination of the effective population size information, the results are presented in the form of Figures 4 and 5 for generations 70 and 10 to the present generation, respectively, for the 8 studied populations (9 datasets).

In the present study, the Ne values of different Holstein cattle populations in the recent generation ranged from 74 (French Holstein population) to 171 (Polish Holstein population) (Table 3), which approximately conform to the ranges reported in previous research (Hillestad et al. 2014). The corresponding values were in the range of 165–194 for the Norwegian Red breed (Hillestad et al. 2017), 74–79 for the Spanish Holstein, 69–79 for the Holstein‐Friesian (Doekes et al. 2018) and 75–85 for the Jersey (Makanjuola, Miglior, et al. 2020). These values (Table 3) were higher than the results of Makanjuola, Maltecca, et al. (2020), indicating Ne values of 43–66 for the Holstein population, but lower than the Holstein‐Friesian population of the Czech Republic, whose Ne values ranged from 202 (GONE) to 283 (NeEstimator v2), depending on the software (Vostry et al. 2023). Chhotaray et al. (2021) obtained effective population sizes of 0.05, 0.02 and 0.01, as well as 40, 44, 46 and 48 for crossbreed Vrindavani cattle with and without applying different MAFs, respectively, which were lower than the values obtained in the present study for Holstein cattle populations.

## 4 Impact of MAF on LD and Ne Estimates

MAF is a key factor influencing the estimation of linkage disequilibrium (LD) and effective population size (Ne), both of which are fundamental to interpreting genetic diversity and population history. Low‐frequency alleles (typically MAF < 0.05) are more susceptible to genotyping errors and sampling variance, potentially inflating short‐range LD and leading to downward‐biased Ne estimates, particularly in recent generations (Anderson et al. 2010; Weng et al. 2022). However, in the present study, we excluded SNPs with MAF < 0.05 during QC procedures, thereby minimizing such biases and enhancing the robustness of our LD and Ne estimates.

Despite the removal of low‐frequency variants, the overall distribution of MAF across populations remains an important consideration. Differences in local breeding strategies, historical bottlenecks and gene flow can lead to varying MAF spectra, which may, in turn, influence LD decay patterns and effective population size trajectories (Boitard et al. 2016; Zhang et al. 2023). Therefore, some of the observed differences in LD levels and recent Ne trends among Holstein populations could reflect underlying variation in allele frequency structure, shaped by both demographic history and selection intensity.

Overall, the exclusion of rare variants (MAF < 0.05) enhances the accuracy of our estimates while ensuring greater comparability across populations. Nevertheless, the careful evaluation of MAF thresholds remains a critical component in population genomic studies, as it helps distinguish true biological signals from technical artefacts (Hill 2019; Wang et al. 2022). Future studies may benefit from stratified analyses on the basis of MAF classes to further elucidate the relationship between allele frequency dynamics, LD structure and demographic history in commercial cattle populations.

Qanbari et al. (2010) reported the effective population size in the German Holstein breed as 149 in the previous four generations. Their results demonstrated a gradual decrease in Ne in recent generations. This value was within the range of data obtained for the previous four generations of populations, from 81 for the Chinese population to 218 for the Swedish population. In another study, using pedigree information, they investigated the potential effects of selection on a closed nucleus of American Angus cattle and an open herd of Brazilian Nelore cattle. They reported the effective population size calculated for the Angus breed through individual increase of inbreeding and the effective size computed through individual increase of the mean Ne in inbreeding as 26 and 29, respectively, which were lower than the values obtained in the present study. The above values were estimated as 58 and 103 for the Nelore breed, respectively. The reason for the large difference between the estimates in the two mentioned methods in Niluri cattle was stated to be the shallow pedigree of the herd because if the pedigree information is incomplete, the calculated inbreeding will have a low bias, and the obtained effective size will be overestimated (Gutiérrez et al. 2009).

The highest and lowest estimated effective population sizes in the past 2000 generations were related to the Irish (*N* = 8803) and Chinese (*N* = 2121) populations, respectively. Moreover, Ne's highest and lowest estimated numerical values for the current generation belonged to the Polish (*N* = 171) and French (*N* = 74) populations, respectively. On the basis of the findings, the fluctuation of numerical values in older generations was high due to the small number of data for distances less than 17 kbp, and consequently, it had a lower reliability. This factor is particularly true in cases where the marker density of the array utilized for sequencing was lower. In any case, the information for recent generations is highly reliable, considering that the result of averaging a large number of data is available for estimation (Mokhber, Shahrbabak, et al. 2019). The declining trend in effective population size (Ne) observed across all studied Holstein populations, particularly the sharp drop over the last 70 generations (Figures 4 and 5), aligns with evidence from recent global studies. Similar reductions have been reported in Holstein populations from diverse regions, including the Czech Republic (Čechová et al. 2023), Ecuador (Cartuche‑Macas et al. 2025) and Nordic countries (Sørensen et al. 2024). These widespread declines are primarily attributed to intensive selection pressure and the use of a limited number of elite sires, resulting in reduced genetic diversity and increased relatedness within populations.

### 4.1 Environmental and Managerial Influences on Ne and LD Variation

Beyond genetic and demographic determinants, cross‐country differences in environmental conditions and management practices likely contributed to the observed heterogeneity in effective population size (Ne) and linkage disequilibrium (LD) patterns. As highlighted in recent studies, intensive breeding programs that rely heavily on a few elite sires via artificial insemination are a primary driver of reduced Ne and increased genetic drift, as observed in populations like France (Waples 2024; Šaran et al. 2023). The considerably larger sample size from China (*n* = 1508) provides more precise estimates of Ne and LD decay compared to smaller cohorts like Sweden (*n* = 23), underscoring the impact of sampling on parameter accuracy (Wang et al. 2022; Waples et al. 2025). Furthermore, local adaptation to climatic stressors, disease prevalence and divergent breeding goals impose unique selective pressures that shape genome‐wide patterns of diversity. For instance, the high Ne estimate in Poland may reflect intentional outcrossing or the incorporation of imported genetic resources to maintain diversity, a strategy that can mitigate the decline in effective population size (Vostry et al. 2023; Ruiz‐López et al. 2024; Salehi et al. 2025). The markedly slower LD decay in the large Chinese cohort, compared to other populations, warrants further investigation into its distinct breeding history, management strategies and potential isolation.

Our findings echo these patterns and correspond with previous research on other breeds. For example, Cho et al. (2012) documented a marked Ne decrease in Hanwoo cattle, from approximately 1150 individuals in distant generations to 84 currently, largely due to widespread artificial insemination practices concentrating genetics among few bulls. Likewise, Sarviaho et al. (2023) observed a reduction in Ne in Finnish Ayrshire cattle, from a peak of 160 individuals in 2011 to about 150, accompanied by shortened generation intervals through genomic selection.

In the Holstein populations analysed in this study, current Ne estimates ranged from 74 in the French population to 171 in the Polish population, values consistent with those reported in major dairy‐producing countries such as Australia, Canada, Denmark, Spain, Ireland and the United States (Doekes et al. 2018). Despite the global abundance of Holstein cattle, these relatively low Ne values reflect extensive use of a narrow genetic base and high genetic relatedness worldwide (Hill and Weir 2011).

It is important to note that although LD‐based Ne estimates are robust for livestock populations (Waples et al. 2025), caution is warranted in interpretation due to potential influences of marker density, MAF filtering and sampling design. In this study, strict filtering (MAF > 0.05) was applied to enhance reliability.

Although current Ne values remain above the critical threshold of 100 recommended to minimize genetic drift and loss of diversity (Meuwissen 2009), the steep recent declines, especially in some populations, are a cause for concern. Such trends highlight the urgent need for effective genetic management strategies to sustain diversity and population viability (Mokhber, Moradi Shahre Babak, et al. 2019; Zhang et al. 2023).

To mitigate these risks, we recommend the adoption of breeding programs that incorporate increased sire diversity, genomic‐assisted mating designs that minimize inbreeding and routine genomic monitoring of Ne to allow timely adjustments. These strategies are essential to balance genetic gain with conservation of genetic variation, ensuring the long‐term sustainability of Holstein populations (Meuwissen et al. 2020; Sørensen et al. 2024).

### 4.2 Complementary Insights From Runs of Homozygosity (ROH)

Although LD‐based Ne estimates are powerful for inferring historical demographic trends, analysing ROH offers a direct and complementary method for quantifying recent inbreeding and genetic bottlenecks, as suggested by the reviewers. ROH segments are contiguous homozygous tracts inherited identical‐by‐descent from a common ancestor, providing a precise measure of genomic autozygosity and recent consanguinity (Ceballos et al. 2018; Kolpakov et al. 2024). Integrating ROH analysis with our LD‐based Ne estimates would provide a multi‐temporal perspective on population history. We would expect populations with low recent Ne (e.g., France_HD: 74) to exhibit a higher burden of long ROH, indicative of recent inbreeding due to the intensive use of a few related sires (Szmatoła et al. 2024; Peripolli et al. 2023). Conversely, populations with larger Ne (e.g., Poland: 171) should display a higher frequency of shorter ROH segments, reflecting a broader genetic base and more distant common ancestors (Meyermans et al. 2020; Mészárosová et al. 2021). Recent advancements in ROH analysis, as demonstrated in Holstein cattle (Šaran et al. 2023; Smaragdov 2025), allow for the identification of selection signatures and genomic regions depleted of heterozygosity. Therefore, a combined approach using both LD decay and genome‐wide ROH metrics is highly recommended for future studies to achieve a comprehensive characterization of historical demography and contemporary inbreeding, ultimately providing a robust toolkit for sustainable genetic management and conservation (Passafaro et al. 2024).

## 5 Conclusion

This study evaluated linkage disequilibrium (LD) and effective population size (Ne) across eight Holstein cattle populations using HD SNP data. The results demonstrate a historical decline in Ne across all populations, with a pronounced reduction observed over the past 70 generations. This decline is attributed to intensive selection, limited sire usage and modern reproductive technologies. Despite the global abundance of Holstein cattle, the genetic foundation of many national subpopulations remains vulnerable due to a narrow genetic base and shared ancestry. However, our findings indicate a recent stabilization or slight recovery in Ne over the past 10 generations in some populations (e.g., Poland, France and the Netherlands). This trend is likely associated with increased genetic material exchange and more informed breeding practices.

Maintaining or increasing Ne is crucial for reducing the risk of genetic drift, preserving allelic diversity and ensuring long‐term population adaptability. These results underscore the need for targeted breeding programs that minimize the overuse of elite sires, promote within‐population diversity and support controlled international genetic exchange. Continuous monitoring of dynamic population trends using LD‐based Ne estimates is recommended to refine breeding decisions.

## Author Contributions

Ronak Salehi, Arash Javanmard and Mahdi Mokhber were responsible for the methodology and study design. Mahdi Mokhber and Ronak Salehi conducted the data analysis. Arash Javanmard and Mahdi Mokhber prepared the original draft version of the manuscript. The manuscript was reviewed and edited by Arash Javanmard, Mahdi Mokhber and Sadegh Alijani. The visualization (tables and figures) was prepared by Ronak Salehi. Arash Javanmard was responsible for supervision and fundraising.

## Funding

The research was funded by Grant: 13/419596/1 of University of Tabriz.

## Ethics Statement

The study was carried out on the farm under standard rearing conditions and in accordance with Slovenian laws. The approval of an ethical review body was not required for this study.

## Conflicts of Interest

The authors declare no conflicts of interest.

## Peer Review

The peer review history for this article is available at https://doi.org/10.1002/vms3.70684.

## Data Availability

The datasets used and/or analysed during the current study are available from the corresponding author on reasonable request.
